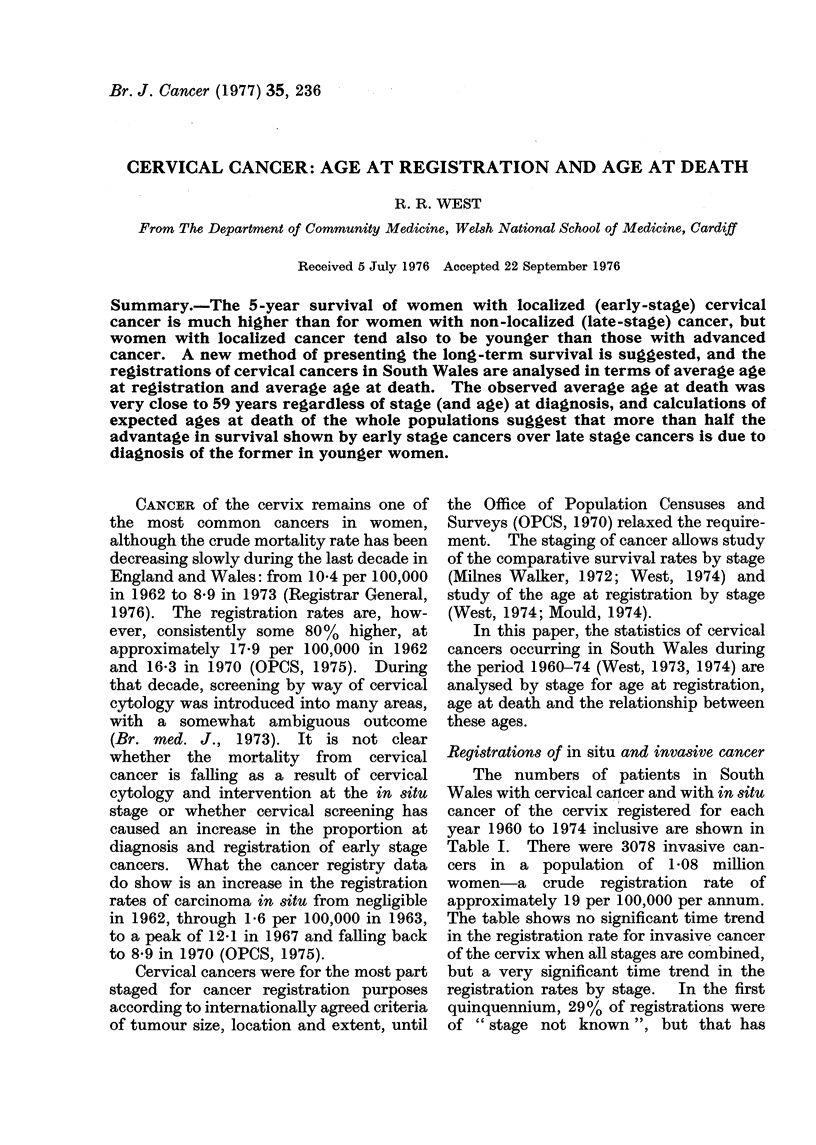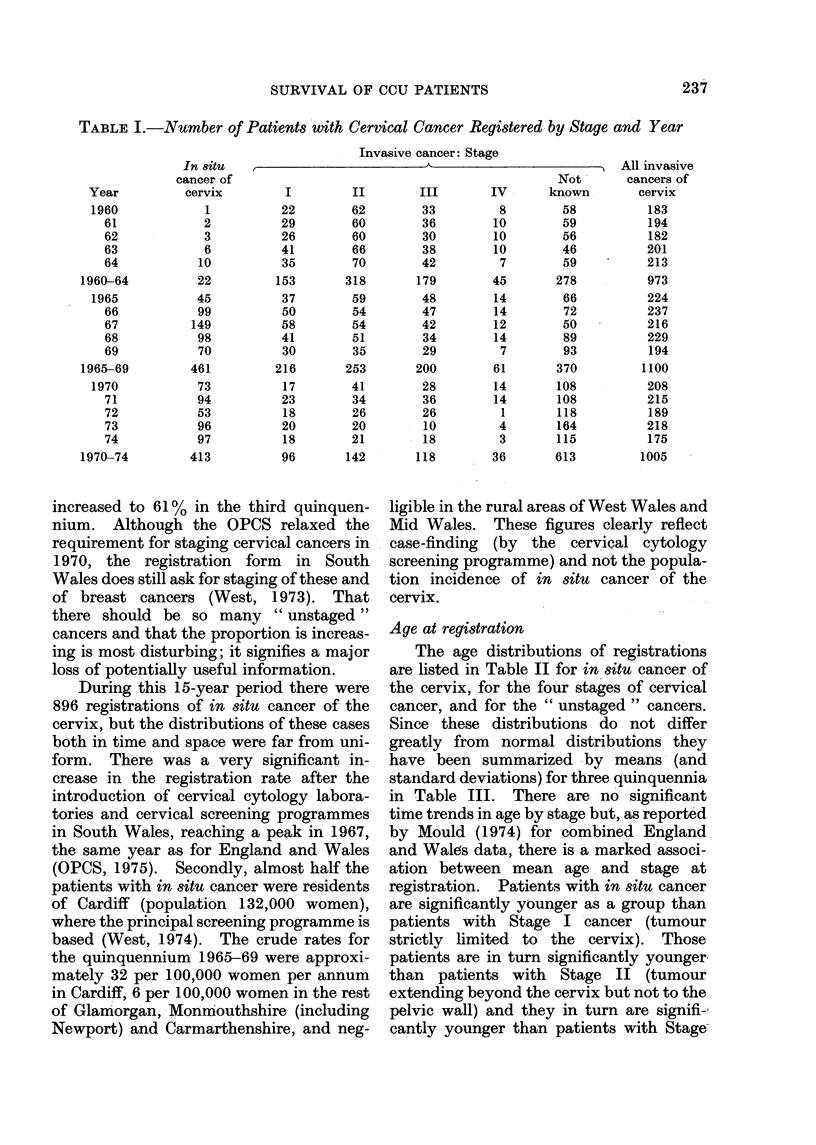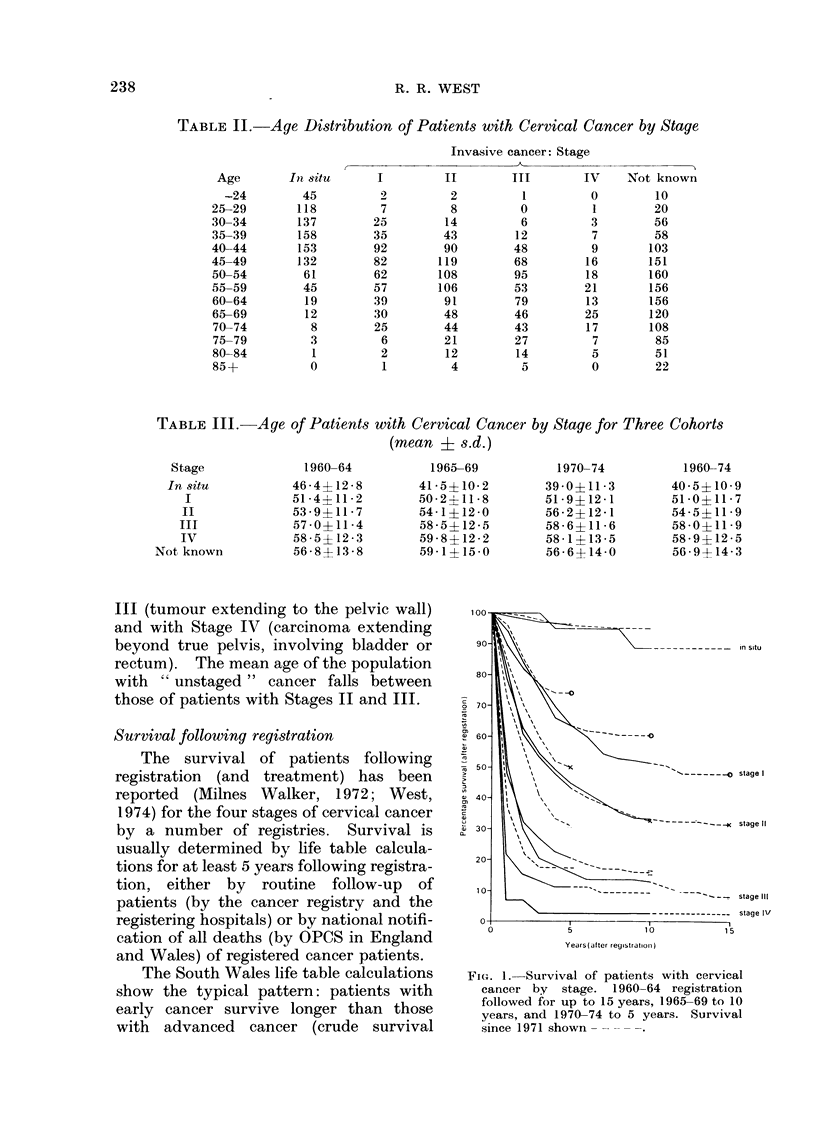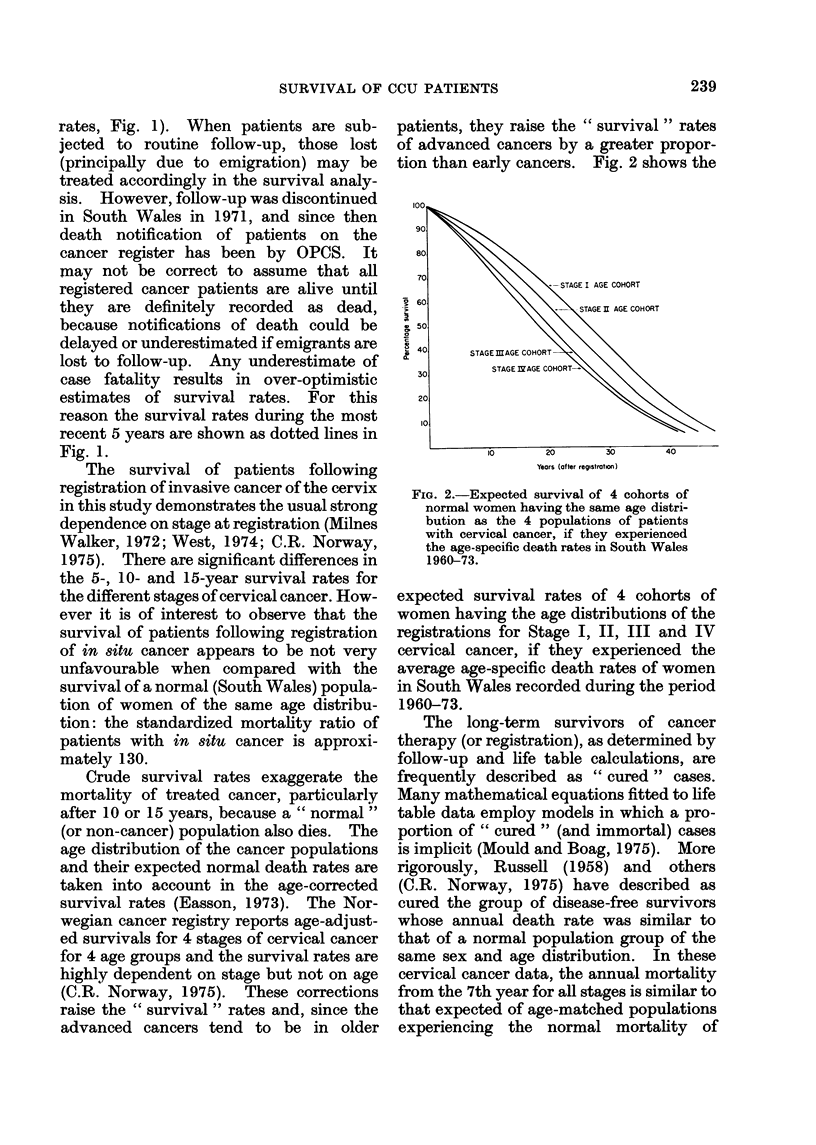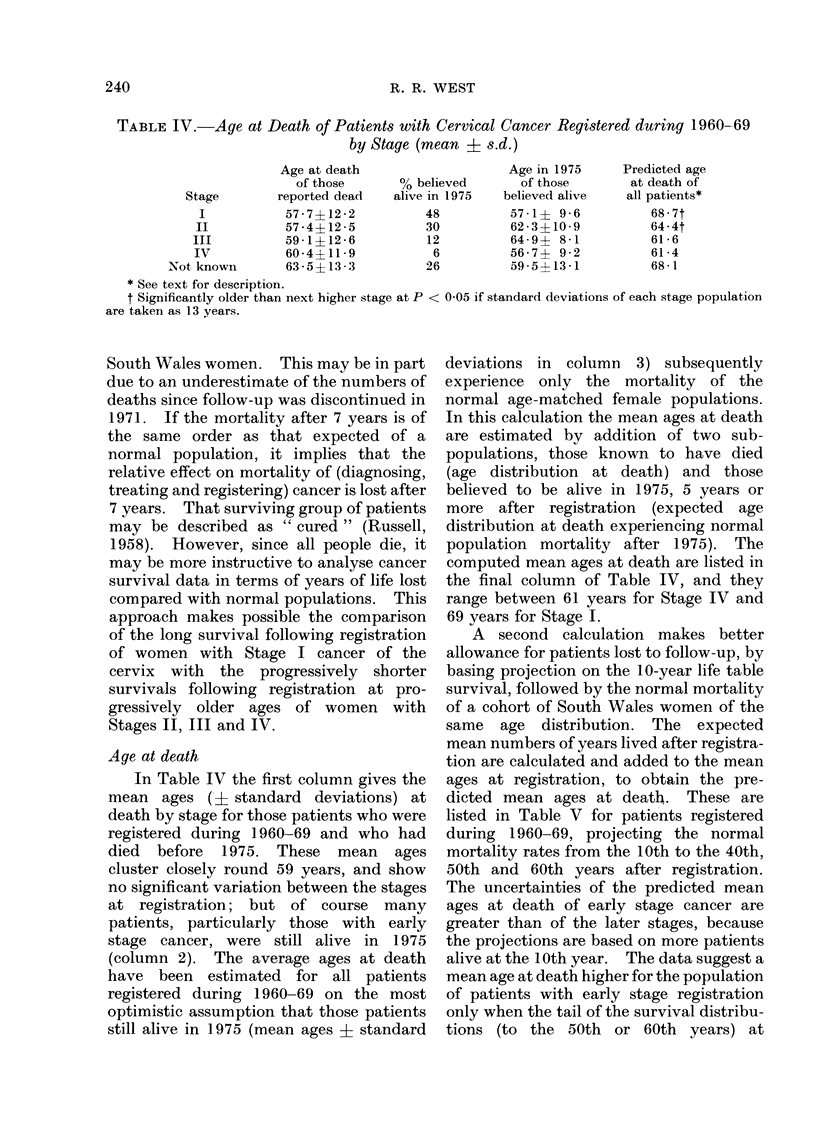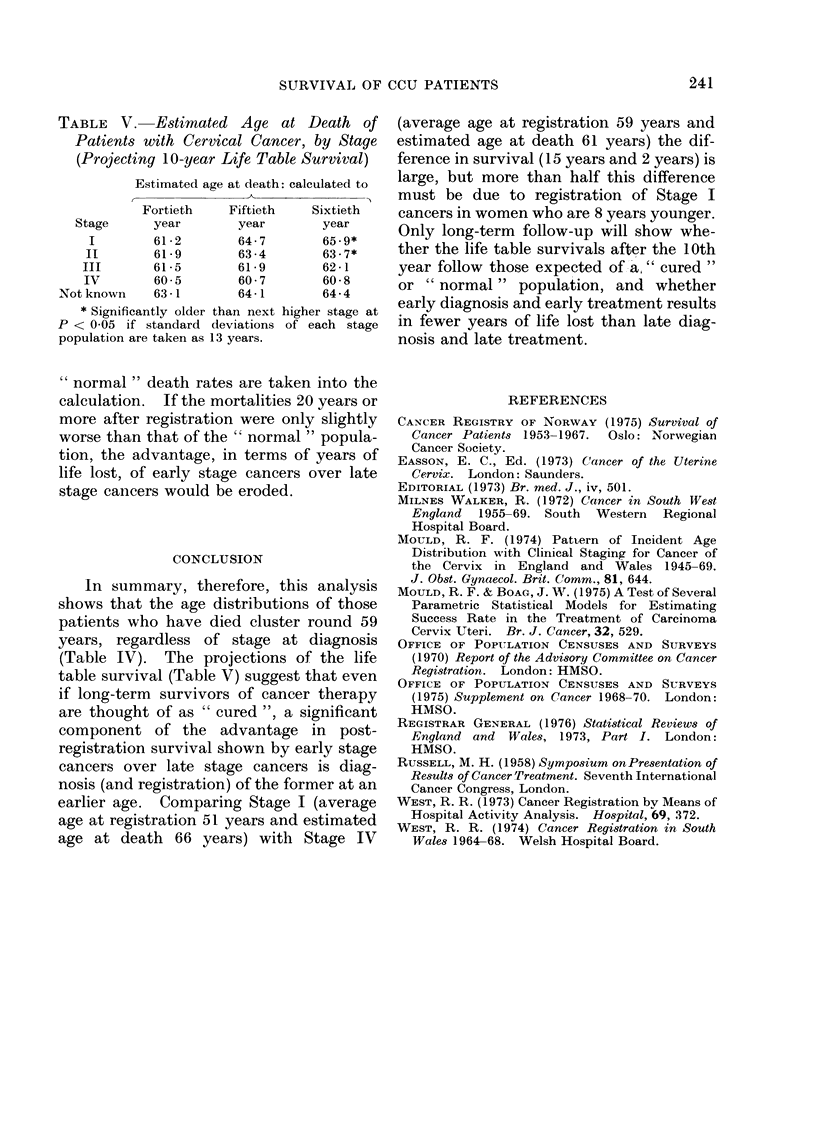# Cervical cancer: Age at registration and age at death.

**DOI:** 10.1038/bjc.1977.32

**Published:** 1977-02

**Authors:** R. R. West

## Abstract

The 5-year survival of women with localized (early-stage) cervical cancer is much higher than for women with non-localized (late-stage) cancer, but women with localized cancer tend also to be younger than those with advanced cancer. A new method of presenting the long-term survival is suggested, and the registrations of cervical cancers in South Wales are analysed in terms of average age at registration and average age at death. The observed average age at death was very close to 59 years regardless of stage (and age) at diagnosis, and calculations of expected ages at death of the whole populations suggest that more than half the advantage in survival shown by early stage cancers over late stage cancers is due to diagnosis of the former in younger women.


*Br. J. Cancer* (1977) **35**, 236

### CERVICAL CANCER: AGE AT REGISTRATION AND AGE AT DEATH

R. R. WEST

*From The Department of Community Medicine, Welsh National School of Medicine, Cardiff*

Received 5 July 1976 Accepted 22 September 1976

**Summary.—The 5-year survival of women with localized (early-stage) cervical cancer is much higher than for women with non-localized (late-stage) cancer, but women with localized cancer tend also to be younger than those with advanced cancer. A new method of presenting the long-term survival is suggested, and the registrations of cervical cancers in South Wales are analysed in terms of average age at registration and average age at death. The observed average age at death was very close to 59 years regardless of stage (and age) at diagnosis, and calculations of expected ages at death of the whole populations suggest that more than half the advantage in survival shown by early stage cancers over late stage cancers is due to diagnosis of the former in younger women.**

CANCER of the cervix remains one of the most common cancers in women, although the crude mortality rate has been decreasing slowly during the last decade in England and Wales: from 10·4 per 100,000 in 1962 to 8·9 in 1973 (Registrar General, 1976). The registration rates are, however, consistently some 80% higher, at approximately 17·9 per 100,000 in 1962 and 16·3 in 1970 (OPCS, 1975). During that decade, screening by way of cervical cytology was introduced into many areas, with a somewhat ambiguous outcome (*Br. med. J.*, 1973). It is not clear whether the mortality from cervical cancer is falling as a result of cervical cytology and intervention at the *in situ* stage or whether cervical screening has caused an increase in the proportion at diagnosis and registration of early stage cancers. What the cancer registry data do show is an increase in the registration rates of carcinoma *in situ* from negligible in 1962, through 1·6 per 100,000 in 1963, to a peak of 12·1 in 1967 and falling back to 8·9 in 1970 (OPCS, 1975).

Cervical cancers were for the most part staged for cancer registration purposes according to internationally agreed criteria of tumour size, location and extent, until the Office of Population Censuses and Surveys (OPCS, 1970) relaxed the requirement. The staging of cancer allows study of the comparative survival rates by stage (Milnes Walker, 1972; West, 1974) and study of the age at registration by stage (West, 1974; Mould, 1974).

In this paper, the statistics of cervical cancers occurring in South Wales during the period 1960–74 (West, 1973, 1974) are analysed by stage for age at registration, age at death and the relationship between these ages.

*Registrations of* in situ *and invasive cancer*

The numbers of patients in South Wales with cervical cancer and with *in situ* cancer of the cervix registered for each year 1960 to 1974 inclusive are shown in Table I. There were 3078 invasive cancers in a population of 1·08 million women—a crude registration rate of approximately 19 per 100,000 per annum. The table shows no significant time trend in the registration rate for invasive cancer of the cervix when all stages are combined, but a very significant time trend in the registration rates by stage. In the first quinquennium, 29% of registrations were of "stage not known", but that has

TABLE I.—*Number of Patients with Cervical Cancer Registered by Stage and Year*

| Year | *In situ* cancer of cervix | Invasive cancer: Stage I | II | III | IV | Not known | All invasive cancers of cervix |
|---|---|---|---|---|---|---|---|
| 1960 | 1 | 22 | 62 | 33 | 8 | 58 | 183 |
| 61 | 2 | 29 | 60 | 36 | 10 | 59 | 194 |
| 62 | 3 | 26 | 60 | 30 | 10 | 56 | 182 |
| 63 | 6 | 41 | 66 | 38 | 10 | 46 | 201 |
| 64 | 10 | 35 | 70 | 42 | 7 | 59 | 213 |
| 1960–64 | 22 | 153 | 318 | 179 | 45 | 278 | 973 |
| 1965 | 45 | 37 | 59 | 48 | 14 | 66 | 224 |
| 66 | 99 | 50 | 54 | 47 | 14 | 72 | 237 |
| 67 | 149 | 58 | 54 | 42 | 12 | 50 | 216 |
| 68 | 98 | 41 | 51 | 34 | 14 | 89 | 229 |
| 69 | 70 | 30 | 35 | 29 | 7 | 93 | 194 |
| 1965–69 | 461 | 216 | 253 | 200 | 61 | 370 | 1100 |
| 1970 | 73 | 17 | 41 | 28 | 14 | 108 | 208 |
| 71 | 94 | 23 | 34 | 36 | 14 | 108 | 215 |
| 72 | 53 | 18 | 26 | 26 | 1 | 118 | 189 |
| 73 | 96 | 20 | 20 | 10 | 4 | 164 | 218 |
| 74 | 97 | 18 | 21 | 18 | 3 | 115 | 175 |
| 1970–74 | 413 | 96 | 142 | 118 | 36 | 613 | 1005 |

increased to 61% in the third quinquennium. Although the OPCS relaxed the requirement for staging cervical cancers in 1970, the registration form in South Wales does still ask for staging of these and of breast cancers (West, 1973). That there should be so many " unstaged " cancers and that the proportion is increasing is most disturbing; it signifies a major loss of potentially useful information.

During this 15-year period there were 896 registrations of *in situ* cancer of the cervix, but the distributions of these cases both in time and space were far from uniform. There was a very significant increase in the registration rate after the introduction of cervical cytology laboratories and cervical screening programmes in South Wales, reaching a peak in 1967, the same year as for England and Wales (OPCS, 1975). Secondly, almost half the patients with *in situ* cancer were residents of Cardiff (population 132,000 women), where the principal screening programme is based (West, 1974). The crude rates for the quinquennium 1965–69 were approximately 32 per 100,000 women per annum in Cardiff, 6 per 100,000 women in the rest of Glamorgan, Monmouthshire (including Newport) and Carmarthenshire, and negligible in the rural areas of West Wales and Mid Wales. These figures clearly reflect case-finding (by the cervical cytology screening programme) and not the population incidence of *in situ* cancer of the cervix.

*Age at registration*

The age distributions of registrations are listed in Table II for *in situ* cancer of the cervix, for the four stages of cervical cancer, and for the " unstaged " cancers. Since these distributions do not differ greatly from normal distributions they have been summarized by means (and standard deviations) for three quinquennia in Table III. There are no significant time trends in age by stage but, as reported by Mould (1974) for combined England and Wales data, there is a marked association between mean age and stage at registration. Patients with *in situ* cancer are significantly younger as a group than patients with Stage I cancer (tumour strictly limited to the cervix). Those patients are in turn significantly younger than patients with Stage II (tumour extending beyond the cervix but not to the pelvic wall) and they in turn are significantly younger than patients with Stage

TABLE II.—*Age Distribution of Patients with Cervical Cancer by Stage*

| | | Invasive cancer: Stage | | | | |
|---|---|---|---|---|---|---|
| Age | *In situ* | I | II | III | IV | Not known |
| –24 | 45 | 2 | 2 | 1 | 0 | 10 |
| 25–29 | 118 | 7 | 8 | 0 | 1 | 20 |
| 30–34 | 137 | 25 | 14 | 6 | 3 | 56 |
| 35–39 | 158 | 35 | 43 | 12 | 7 | 58 |
| 40–44 | 153 | 92 | 90 | 48 | 9 | 103 |
| 45–49 | 132 | 82 | 119 | 68 | 16 | 151 |
| 50–54 | 61 | 62 | 108 | 95 | 18 | 160 |
| 55–59 | 45 | 57 | 106 | 53 | 21 | 156 |
| 60–64 | 19 | 39 | 91 | 79 | 13 | 156 |
| 65–69 | 12 | 30 | 48 | 46 | 25 | 120 |
| 70–74 | 8 | 25 | 44 | 43 | 17 | 108 |
| 75–79 | 3 | 6 | 21 | 27 | 7 | 85 |
| 80–84 | 1 | 2 | 12 | 14 | 5 | 51 |
| 85+ | 0 | 1 | 4 | 5 | 0 | 22 |

TABLE III.—*Age of Patients with Cervical Cancer by Stage for Three Cohorts (mean ± s.d.)*

| Stage | 1960–64 | 1965–69 | 1970–74 | 1960–74 |
|---|---|---|---|---|
| *In situ* | 46·4±12·8 | 41·5±10·2 | 39·0±11·3 | 40·5±10·9 |
| I | 51·4±11·2 | 50·2±11·8 | 51·9±12·1 | 51·0±11·7 |
| II | 53·9±11·7 | 54·1±12·0 | 56·2±12·1 | 54·5±11·9 |
| III | 57·0±11·4 | 58·5±12·5 | 58·6±11·6 | 58·0±11·9 |
| IV | 58·5±12·3 | 59·8±12·2 | 58·1±13·5 | 58·9±12·5 |
| Not known | 56·8±13·8 | 59·1±15·0 | 56·6±14·0 | 56·9±14·3 |

III (tumour extending to the pelvic wall) and with Stage IV (carcinoma extending beyond true pelvis, involving bladder or rectum). The mean age of the population with "unstaged" cancer falls between those of patients with Stages II and III.

*Survival following registration*

The survival of patients following registration (and treatment) has been reported (Milnes Walker, 1972; West, 1974) for the four stages of cervical cancer by a number of registries. Survival is usually determined by life table calculations for at least 5 years following registration, either by routine follow-up of patients (by the cancer registry and the registering hospitals) or by national notification of all deaths (by OPCS in England and Wales) of registered cancer patients.

The South Wales life table calculations show the typical pattern: patients with early cancer survive longer than those with advanced cancer (crude survival

FIG. 1.—Survival of patients with cervical cancer by stage. 1960–64 registration followed for up to 15 years, 1965–69 to 10 years, and 1970–74 to 5 years. Survival since 1971 shown – – – – –.

rates, Fig. 1). When patients are subjected to routine follow-up, those lost (principally due to emigration) may be treated accordingly in the survival analysis. However, follow-up was discontinued in South Wales in 1971, and since then death notification of patients on the cancer register has been by OPCS. It may not be correct to assume that all registered cancer patients are alive until they are definitely recorded as dead, because notifications of death could be delayed or underestimated if emigrants are lost to follow-up. Any underestimate of case fatality results in over-optimistic estimates of survival rates. For this reason the survival rates during the most recent 5 years are shown as dotted lines in Fig. 1.

The survival of patients following registration of invasive cancer of the cervix in this study demonstrates the usual strong dependence on stage at registration (Milnes Walker, 1972; West, 1974; C.R. Norway, 1975). There are significant differences in the 5-, 10- and 15-year survival rates for the different stages of cervical cancer. However it is of interest to observe that the survival of patients following registration of *in situ* cancer appears to be not very unfavourable when compared with the survival of a normal (South Wales) population of women of the same age distribution: the standardized mortality ratio of patients with *in situ* cancer is approximately 130.

Crude survival rates exaggerate the mortality of treated cancer, particularly after 10 or 15 years, because a " normal " (or non-cancer) population also dies. The age distribution of the cancer populations and their expected normal death rates are taken into account in the age-corrected survival rates (Easson, 1973). The Norwegian cancer registry reports age-adjusted survivals for 4 stages of cervical cancer for 4 age groups and the survival rates are highly dependent on stage but not on age (C.R. Norway, 1975). These corrections raise the " survival " rates and, since the advanced cancers tend to be in older patients, they raise the " survival " rates of advanced cancers by a greater proportion than early cancers. Fig. 2 shows the

Fig. 2.—Expected survival of 4 cohorts of normal women having the same age distribution as the 4 populations of patients with cervical cancer, if they experienced the age-specific death rates in South Wales 1960–73.

expected survival rates of 4 cohorts of women having the age distributions of the registrations for Stage I, II, III and IV cervical cancer, if they experienced the average age-specific death rates of women in South Wales recorded during the period 1960–73.

The long-term survivors of cancer therapy (or registration), as determined by follow-up and life table calculations, are frequently described as " cured " cases. Many mathematical equations fitted to life table data employ models in which a proportion of " cured " (and immortal) cases is implicit (Mould and Boag, 1975). More rigorously, Russell (1958) and others (C.R. Norway, 1975) have described as cured the group of disease-free survivors whose annual death rate was similar to that of a normal population group of the same sex and age distribution. In these cervical cancer data, the annual mortality from the 7th year for all stages is similar to that expected of age-matched populations experiencing the normal mortality of

TABLE IV.—*Age at Death of Patients with Cervical Cancer Registered during* 1960–69 *by Stage* (*mean* ± *s.d.*)

| Stage | Age at death of those reported dead | % believed alive in 1975 | Age in 1975 of those believed alive | Predicted age at death of all patients* |
|---|---|---|---|---|
| I | 57·7±12·2 | 48 | 57·1± 9·6 | 68·7† |
| II | 57·4±12·5 | 30 | 62·3±10·9 | 64·4† |
| III | 59·1±12·6 | 12 | 64·9± 8·1 | 61·6 |
| IV | 60·4±11·9 | 6 | 56·7± 9·2 | 61·4 |
| Not known | 63·5±13·3 | 26 | 59·5±13·1 | 68·1 |

* See text for description.

† Significantly older than next higher stage at $P < 0{\cdot}05$ if standard deviations of each stage population are taken as 13 years.

South Wales women. This may be in part due to an underestimate of the numbers of deaths since follow-up was discontinued in 1971. If the mortality after 7 years is of the same order as that expected of a normal population, it implies that the relative effect on mortality of (diagnosing, treating and registering) cancer is lost after 7 years. That surviving group of patients may be described as " cured " (Russell, 1958). However, since all people die, it may be more instructive to analyse cancer survival data in terms of years of life lost compared with normal populations. This approach makes possible the comparison of the long survival following registration of women with Stage I cancer of the cervix with the progressively shorter survivals following registration at progressively older ages of women with Stages II, III and IV.

*Age at death*

In Table IV the first column gives the mean ages (± standard deviations) at death by stage for those patients who were registered during 1960–69 and who had died before 1975. These mean ages cluster closely round 59 years, and show no significant variation between the stages at registration; but of course many patients, particularly those with early stage cancer, were still alive in 1975 (column 2). The average ages at death have been estimated for all patients registered during 1960–69 on the most optimistic assumption that those patients still alive in 1975 (mean ages ± standard deviations in column 3) subsequently experience only the mortality of the normal age-matched female populations. In this calculation the mean ages at death are estimated by addition of two sub-populations, those known to have died (age distribution at death) and those believed to be alive in 1975, 5 years or more after registration (expected age distribution at death experiencing normal population mortality after 1975). The computed mean ages at death are listed in the final column of Table IV, and they range between 61 years for Stage IV and 69 years for Stage I.

A second calculation makes better allowance for patients lost to follow-up, by basing projection on the 10-year life table survival, followed by the normal mortality of a cohort of South Wales women of the same age distribution. The expected mean numbers of years lived after registration are calculated and added to the mean ages at registration, to obtain the predicted mean ages at death. These are listed in Table V for patients registered during 1960–69, projecting the normal mortality rates from the 10th to the 40th, 50th and 60th years after registration. The uncertainties of the predicted mean ages at death of early stage cancer are greater than of the later stages, because the projections are based on more patients alive at the 10th year. The data suggest a mean age at death higher for the population of patients with early stage registration only when the tail of the survival distributions (to the 50th or 60th years) at

TABLE V.—*Estimated Age at Death of Patients with Cervical Cancer, by Stage (Projecting 10-year Life Table Survival)*

| | Estimated age at death: calculated to | | |
|---|---|---|---|
| Stage | Fortieth year | Fiftieth year | Sixtieth year |
| I | 61·2 | 64·7 | 65·9* |
| II | 61·9 | 63·4 | 63·7* |
| III | 61·5 | 61·9 | 62·1 |
| IV | 60·5 | 60·7 | 60·8 |
| Not known | 63·1 | 64·1 | 64·4 |

* Significantly older than next higher stage at $P < 0{\cdot}05$ if standard deviations of each stage population are taken as 13 years.

" normal " death rates are taken into the calculation. If the mortalities 20 years or more after registration were only slightly worse than that of the " normal " population, the advantage, in terms of years of life lost, of early stage cancers over late stage cancers would be eroded.

### CONCLUSION

In summary, therefore, this analysis shows that the age distributions of those patients who have died cluster round 59 years, regardless of stage at diagnosis (Table IV). The projections of the life table survival (Table V) suggest that even if long-term survivors of cancer therapy are thought of as " cured ", a significant component of the advantage in post-registration survival shown by early stage cancers over late stage cancers is diagnosis (and registration) of the former at an earlier age. Comparing Stage I (average age at registration 51 years and estimated age at death 66 years) with Stage IV (average age at registration 59 years and estimated age at death 61 years) the difference in survival (15 years and 2 years) is large, but more than half this difference must be due to registration of Stage I cancers in women who are 8 years younger. Only long-term follow-up will show whether the life table survivals after the 10th year follow those expected of a " cured " or " normal " population, and whether early diagnosis and early treatment results in fewer years of life lost than late diagnosis and late treatment.

### REFERENCES

CANCER REGISTRY OF NORWAY (1975) *Survival of Cancer Patients* 1953–1967. Oslo: Norwegian Cancer Society.

EASSON, E. C., Ed. (1973) *Cancer of the Uterine Cervix.* London: Saunders.

EDITORIAL (1973) *Br. med. J.*, iv, 501.

MILNES WALKER, R. (1972) *Cancer in South West England* 1955–69. South Western Regional Hospital Board.

MOULD, R. F. (1974) Pattern of Incident Age Distribution with Clinical Staging for Cancer of the Cervix in England and Wales 1945–69. *J. Obst. Gynaecol. Brit. Comm.*, **81**, 644.

MOULD, R. F. & BOAG, J. W. (1975) A Test of Several Parametric Statistical Models for Estimating Success Rate in the Treatment of Carcinoma Cervix Uteri. *Br. J. Cancer*, **32**, 529.

OFFICE OF POPULATION CENSUSES AND SURVEYS (1970) *Report of the Advisory Committee on Cancer Registration.* London: HMSO.

OFFICE OF POPULATION CENSUSES AND SURVEYS (1975) *Supplement on Cancer* 1968–70. London: HMSO.

REGISTRAR GENERAL (1976) *Statistical Reviews of England and Wales*, 1973, *Part I.* London: HMSO.

RUSSELL, M. H. (1958) *Symposium on Presentation of Results of Cancer Treatment.* Seventh International Cancer Congress, London.

WEST, R. R. (1973) Cancer Registration by Means of Hospital Activity Analysis. *Hospital*, **69**, 372.

WEST, R. R. (1974) *Cancer Registration in South Wales* 1964–68. Welsh Hospital Board.